# Excitation of Surface Plasmon Polariton Modes with Double-Layer Gratings of Graphene

**DOI:** 10.3390/nano12071144

**Published:** 2022-03-30

**Authors:** Jianping Liu, Weilin Wang, Fang Xie, Xiaoming Zhang, Xia Zhou, Yijun Yuan, Lingling Wang

**Affiliations:** 1 College of Physics Science and Engineering Technology, Yichun University, Yichun 336000, China; wangwl26@163.com (W.W.); xiefang2005@163.com (F.X.); yijunyuan@126.com (Y.Y.); 2 College of Literature, Journalism and Communication, Yichun University, Yichun 336000, China; zhouxia_2003@126.com; 3 Laboratory for Micro-Nano Physics and Technology of Hunan Province, School of Physics and Electronics, Hunan University, Changsha 410082, China; llwang@hnu.edu.cn

**Keywords:** double-layer grating, graphene, surface plasmon polariton, waveguide

## Abstract

A long-range surface plasmon polariton (SPP) waveguide, composed of double-layer graphene, can be pivotal in transferring and handling mid-infrared electromagnetic waves. However, one of the key challenges for this type of waveguide is how to excite the SPP modes through an incident light beam. In this study, our proposed design of a novel grating, consisting of a graphene-based cylindrical long-range SPP waveguide array, successfully addresses this issue using finite-difference time-domain simulations. The results show that two types of symmetric coupling modes (SCMs) are excited through a normal incident light. The transmission characteristics of the two SCMs can be manipulated by changing the interaction of the double-layer gratings of graphene as well as by varying various parameters of the device. Similarly, four SCMs can be excited and controlled by an oblique incident light because this light source is equivalent to two orthogonal beams of light. Furthermore, this grating can be utilized in the fabrication of mid-infrared optical devices, such as filters and refractive index sensors. This grating, with double-layer graphene arrays, has the potential to excite and manipulate the mid-infrared electromagnetic waves in future photonic integrated circuits.

## 1. Introduction

Surface plasmon polaritons (SPPs) [1], which are the collective oscillations of electrons at the interface between a conductor and a dielectric, are promising candidates that can be used to overcome the diffraction limit of light owing to their strong sub-wavelength confinement of electromagnetic (EM) waves. Noble metals are considered the most promising SPP materials [2]. Therefore, various noble metal-based optical devices [3,4,5,6,7] have been studied in the visible range of light for future photonic integrated circuits. However, in the mid-infrared range, noble metal-based optical devices exhibit significantly weak EM field confinement and strong transmission losses, making them inefficient in this wavelength range. Therefore, a new SPP material is needed that can be used in mid-infrared range applications, such as spectroscopy, communication, sensing, and homeland security [8].

Graphene [9], a typical two-dimensional material formed by a single layer of carbon atoms packed into a honeycomb lattice, has several distinct advantages over the noble metals in the mid-infrared range [10], such as the deep sub-wavelength confinement of EM waves, lower transmission losses, and graphene-Fermi energy-dependent EM properties [11]. Because of these unique EM properties, various graphene-based optical devices [12,13,14,15] have been proposed in recent years. However, since the wave vector of a graphene SPP wave is considerably larger than that of an incident light beam, exciting an SPP wave in graphene by matching these two wave vectors is significantly challenging. Several methods, such as tailoring graphene into various geometric-patterned nanostructures [16,17,18], near-field probe coupling [19,20], prism coupling [21,22,23], dielectric grating coupling [24,25,26], metal grating coupling [27], and graphene grating coupling [24,28,29,30,31], have been proposed to overcome this mismatch to explore the advantages of graphene further. Among them, graphene grating, consisting of a graphene-based SPP waveguide array placed on a substrate, effectively compensates for this mismatch using the wave vector of the incident light by diffraction. Currently, most graphene gratings are composed of the most common type of SPP waveguides with a single layer of graphene. Obviously, for a newly proposed graphene SPP waveguide with potentially enhanced performance, a novel graphene grating has to be designed to excite the corresponding SPP mode.

Recently, we investigated an intriguing type of graphene-based long-range SPP (LRSPP) waveguide [32,33], that is composed of one or more dielectric layers placed between two layers of graphene. The symmetric coupling modes (SCMs) and the anti-symmetric coupling modes (ASCMs) are transferred through this waveguide when the two graphene layers are effectively coupled. The SCMs exhibit an ultra-strong EM field confinement and short propagation length, whereas the ASCMs exhibit completely opposite characteristics. Thus, the SCMs and the ASCMs can also be named as short-range SPP (SRSPP) and LRSPP modes, respectively. Because of these interesting EM features, this type of waveguide can have a wide range of applications in future photonic integrated circuits [13,15]. However, in the above-mentioned studies [32,33], methods of exciting the two types of modes have not been introduced. In the present study, by using the finite-difference time-domain (FDTD) method, we propose a grating composed of the graphene-based cylindrical LRSPP waveguide array. The results demonstrate that two and four types of SCMs are excited through a normal and an oblique incident light by this grating, respectively. In addition, the transmission characteristics of the SCMs are effectively controlled by changing the interaction of the double-layer grating of graphene as well as by varying the other parameters of the device. Furthermore, we can design some mid-infrared optical devices, such as the filters and the refractive index sensors, using the grating.

## 2. Materials and Methods

**Figure 1:**
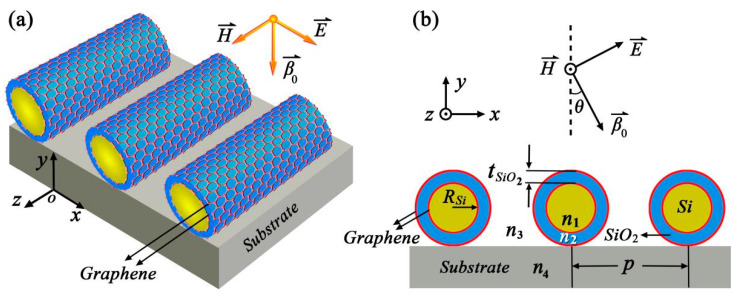
(**a**) Schematic of the proposed grating consisting of the graphene-based cylindrical LRSPP waveguide array. (**b**) Cross-section of the proposed grating.

A schematic diagram of the proposed grating, which is comprised of a graphene LRSPP waveguide array placed on a substrate, is shown in Figure 1a. This waveguide is composed of a cylindrical silicon nanowire core surrounded by an inner graphene layer, a silica layer, and an outer graphene layer as shown in Figure 1b. The radius of the nanowire and the thickness of the silica layer are labeled as *R_Si_* and *t*_*SiO*_2__, respectively. To fabricate such a grating, one of the key challenges is how to obtain a feasible manufacturing technique for the cylindrical LRSPP waveguide. Li et al. [34] have reported how to transfer a graphene flake onto a microfiber in detail. Several experiments [35,36] have shown that the dielectric nanowire can be tightly surrounded by the graphene layer because of the van der Waals force. Thus, this waveguide can be formed by rolling a graphene flake around the dielectric nanowire from the inner to the outer layer, step by step. The silica layer can be formed using plasma-enhanced chemical vapor deposition (PECVD) technology and the thickness of the silica layer can be controlled by tuning the deposition conditions [37]. For simplicity, we have not considered the dispersive characteristics of the medium in this paper. The refractive indexes of the silicon nanowire and the silica layer are set as *n*_1_ = *n_Si_* = 3.455 and *n*_2_ = *n*_*SiO*_2__ = 1.445, respectively. We also assume that this device is embedded in a uniform silica environment with a refractive index of *n*_3_ = *n*_4_ = *n*_*SiO*_2__ = 1.445. The period of the waveguide array is labeled as *p*. The permittivity of graphene is expressed as a complex permittivity with a surface-normal effective permittivity of 2.5 and an in-plane effective permittivity of 2.5 + *iσ_g_/(ε*_0_*ωd)* [38,39], where *σ_g_* is the conductivity of graphene, *ε*_0_ is the permittivity of free space, *ω* is the angular frequency of the incident light, and *d* is the thickness of a graphene layer. Using the Drude-like formula [38,40], *σ_g_* is expressed as *σ_g_(ω)* = *ie*^2^*E_f_*/[*πħ*^2^(*ω + iτ*^−1^)], where *e* is the electron charge, *E_f_* is the Fermi energy, and *τ* is the electron relaxation time. The latter is calculated from the relation *τ* = *μE_f_*/(*ev_F_*^2^), where *μ* is the electron mobility, and *v_F_* = 10^−6^ ms^−1^ is the Fermi velocity. In this paper, unless stated otherwise, the following values of different parameters are used: *μ* = 1.0 m^2^/(Vs), *E_f_* = 0.8 eV, *T* = 300 K, and *d* = 1 nm.

In this study, we employed the commercial software of the Lumerical FDTD Solutions to investigate the features of the proposed grating. The two-dimensional simulation was performed for a single unit of the grating in *xy*-plane. The simulation region was truncated by periodic boundaries in the *x*-direction and perfectly matched layer boundaries in the *y*-direction. The mesh sizes were set as 0.1 nm along the *x*, *y* axes inside the grating unit, and gradually increased outside the grating unit.

## 3. Transmission Characteristics of This Grating with a Perpendicular Incident Light

First, a normal incident plane wave (*θ* = 0°) was projected onto the graphene LRSPP waveguide array along the negative *y*-direction. *θ* represents the angle between the direction of $\vec{\beta }$ and the negative *y*-direction, in which $\vec{\beta }$ is the propagation constant of the incident light. We investigated the physical mechanism of exciting the SPP modes through the grating. It is widely known that SPP oscillations are excited in a grating consisting of a graphene waveguide array [24,28], when the electric field polarization is perpendicular to the grating. Thus, the SPP modes can also be excited by transverse electric (TE) and transverse magnetic (TM) waves. In this study, when the SPP oscillations are excited by a plane wave, a sharp notch corresponding to the resonant frequency is observed in the transmission spectrum. This resonant behavior has been demonstrated experimentally in a graphene ribbon grating [41]. A similar physical phenomenon is also observed in our proposed grating using numerical simulation. To excite such an SPP wave in graphene with a free-space optical wave, their large difference of wave-vector has to be overcome. Using a grating is an effective way to compensate for the wave-vector mismatch. The wave-vector matching equation of this grating can be expressed as

(1) $$k_{0}\sin\theta +mk_{grating}=k_{spp}$$

where *k*_0_ = 2π/*λ*_0_ is the *k*-vector of the incident light, *k_grating_* is the compensatory *k*-vector by the grating, *k_spp_* is the *k*-vector of the excited SPP mode, and *m* is the diffraction order. Due to *k_spp_* = *n_r_k*_0_, the Equation (1) can be expressed approximatively as

(2) $$\frac{2\pi }{\lambda_{0}}\sin\theta +m\frac{2\pi }{p}=n_{r}\frac{2\pi }{\lambda_{0}},$$

where *n_r_* is the real part of the effective refractive index of the excited SPP mode. However, as the graphene LRSPP waveguide has two graphene layers, our proposed grating is equivalent to two optical gratings. The first grating consists of the inner layer graphene array and the silicon nanowire array, whereas the second grating consists of the outer layer graphene array, the silica layer array, and the silicon nanowire array. For simplicity, the first and the second gratings are referred to as the inner and the outer gratings, respectively. Either of them can individually excite SPP resonant modes. However, according to Equation (2), their corresponding resonant wavelength is different for the same incident light, because the SPP mode excited by two gratings has a different *n_r_* value.

**Figure 2:**
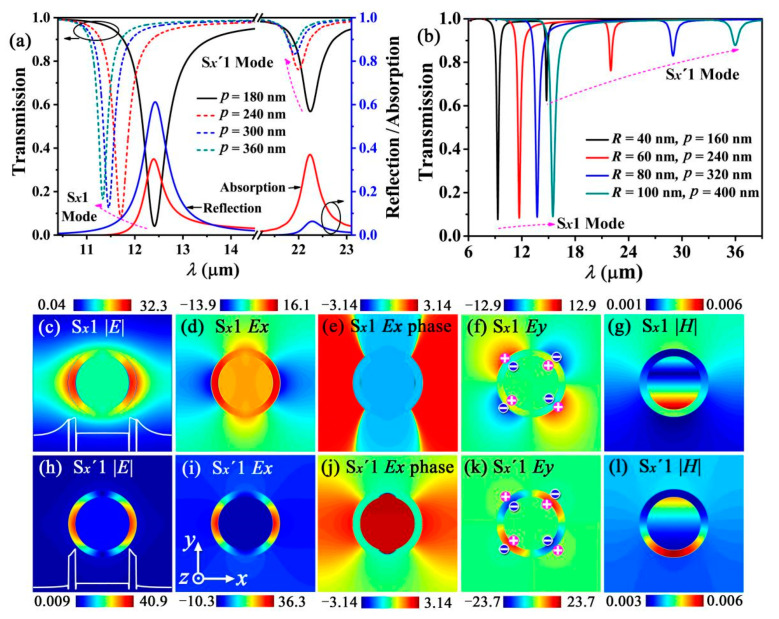
(**a**) Simulated transmission spectra with different periods of waveguide array *p* for a fixed waveguide radius *R =* 60 nm. The blue solid line and the red solid line represent the reflection spectra and absorption spectra as *p* = 180 nm, respectively. (**b**) Simulated transmission spectra with different waveguide radius *R* and period of waveguide array *p* following the relation *p* = 4*R*. (**c**–**g**) show the |*E*|, *E_x_*, *E_x_* phase, *E_y_*, and |*H*| distribution of the S*_x_*1 mode on the cross-section of the waveguide, respectively. (**h**–**l**) show the |*E*|, *E_x_*, *E_x_* phase, *E_y_*, and |*H*| distribution of the S*_x_*′1 mode on the cross-section of the waveguide, respectively. The white lines in (**c**,**h**) describe the |*E|* distribution of the S*_x_*1 and S*_x_*′1 modes along the *x*-axis of the waveguide, respectively. The “+” and “−” symbols in (**f**,**k**) represent the positive and negative charges, respectively. This charge distribution is obtained from the EM field boundary conditions of $\vec{n}\cdot ({\vec{D}}_{2}-{\vec{D}}_{1})=\sigma$.

Figure 2a shows that two main SPP resonant modes are excited by a normal incident plane EM wave with an electric field polarized along the *x*-direction. Both modes are 1-order SCMs because their *E_x_* and *E_x_* phase distributions are symmetric with respect to the *y*-axis (Figure 2d,e,i,j). The SCMs at the shorter and longer wavelengths are referred to as the S*_x_*1 mode and S*_x_*′1 modes, respectively. Both the S*_x_*1 and S*_x_*′1 modes are neither TE modes nor TM modes, according to the work described in [33]. In addition, Figure 2a also shows that the S*_x_*′1 mode corresponds to stronger transmission and weaker reflection than the S*_x_*1 mode. However, the two modes have nearly identical absorptions.

The EM field energy of the S*_x_*′1 mode is effectively confined between the two graphene layers (Figure 2h,l), whereas that of the S*_x_*1 mode is not strictly confined between the two graphene layers and spreads outside the waveguide, gradually decaying with increasing distance from the waveguide (Figure 2c,g). As evident from Figure 2c,g,h,l, the S*_x_*1 and S*_x_*′1 modes are excited by the outer and the inner gratings, respectively, which results in the contrasting EM field energies of the two modes. In addition, there is an interaction between the two gratings. For example, when the S*_x_*′1 mode is excited, the EM oscillations of the quadrupoles occur in the inner grating, which further induces quadrupole oscillations with an opposite charge distribution in the outer grating (Figure 2k). This interaction causes a shift in the resonant wavelength of the S*_x_*′1 mode. A similar EM oscillations of the quadrupoles occur in the outer grating (Figure 2f), and a similar shift in the resonant wavelength of the S*_x_*1 mode is caused by the interaction between the two gratings. Therefore, the resonant wavelengths of the two modes are determined by the individual characteristics of the gratings as well as by the interaction between the two gratings.

Based on the above principle of double graphene gratings, we investigated the dependence of the resonant wavelength on various geometrical parameters of this waveguide. For convenience, we defined the equivalent radii of the inner and outer gratings, *R_i,_* and *R_o_*, as *R_i_* = *R_Si_* + *d* and *R_o_* = *R_Si_* + *t*_*SiO*_2__ + 2*d*, respectively. Therefore, *R_o_* = *R*, where *R* is the total radius of the graphene LRSPP waveguide. We further defined the occupation rate of the inner and outer gratings, *η_i_* and *η_o_*, as *η_i_* = 2*R_i_/p* and *η_o_* = 2*R_o_/p*, respectively. The parameters of this device can be effectively estimated by combining Equation (2) and the result described in [33]. Then, we assumed that *R_Si_* = 46 nm and *t*_*SiO*_2__ = 12 nm, and increased *p* from 180 nm to 360 nm. In this case, *R_i_* and *R_o_* are constants, whereas *η_i_* and *η_o_* decrease. According to previous reports [24,28,30], a change in the occupation rate of a grating significantly changes the coupling strength between the components of the grating and subsequently causes a considerable shift in the resonant wavelength. Although establishing a mathematical relation between the resonant wavelength and the occupation rate is difficult, several studies [24,28,30] qualitatively demonstrated that the resonant wavelength decreases (blue shift) with decreasing occupation rate. Our study verifies this feature. One can find that the resonant wavelengths of the S*_x_*1 and S*_x_*′1 modes decrease with increasing *p* because *η_i_* and *η_o_* decrease in this case (Figure 2a).

Next, we fixed *t*_*SiO*_2__ as 12 nm and increased the *R_Si_* and *p* values, while simultaneously ensuring that *η_o_* was fixed at 0.5 (i.e., *p* = 4*R*). However, *η_i_* increases with increasing *p* because *η_i_* = 2*R_i_*/*p* = 0.5 − 2(*t*_*SiO*_2__ + *d*)/*p*. Thus, the resonant wavelength of the S*_x_*′1 mode increases (red shift). Further, *R_i_* and *R_o_* increase with increasing *R_Si_*. Therefore, a longer wavelength is required to wrap the waveguide when same-order modes are excited. We have derived an equation to calculate the resonant frequency for the grating formed via cylindrical graphene-coated nanowire array (GCNA) [28]. According to the equation, the resonant frequency, *ω_p_*, is inversely proportional to the square root of the radius of the cylindrical graphene waveguide, *r*, (i.e., $\;\omega_{p}\propto \sqrt{1/r}$). This subsequently implies that the resonant wavelength, *λ*, is directly proportional to the square root of *r* (i.e., $\lambda \propto \sqrt{r}$). Our proposed double layers of gratings are similar to two GCNAs. Figure 2b shows that the resonant wavelengths of the S*_x_*1 and S*_x_*′1 modes increase with increasing *R* (red shift). Moreover, the red shift magnitudes of the S*_x_*′1 mode are significantly larger than those of the S1 modes, because the red shift in the S*_x_*′1 mode originates not only from increasing *R* but also from increasing *η_i_*.

**Figure 3:**
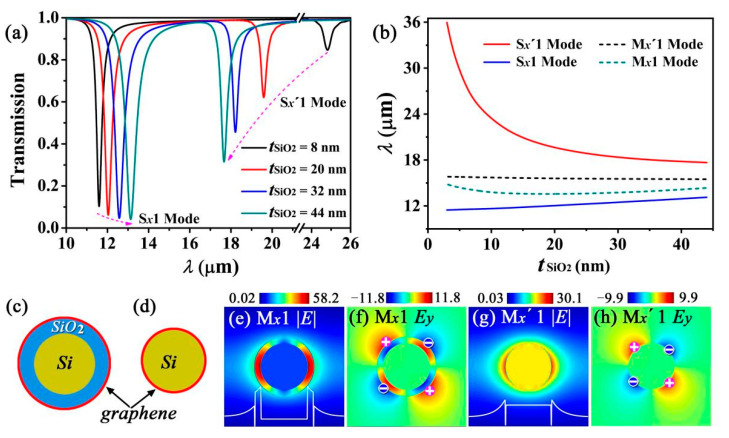
(**a**) Simulated transmission spectra with different *t*_*SiO*_2__. (**b**) Resonant wavelength as a function of *t*_*SiO*_2__ for different modes. (**c**,**d**) show the schematics of the components of the graphene hybrid waveguide array (GHWA) and the graphene-coated nanowire array (GCNA) gratings, respectively. (**e**,**f**) show the |*E*| and *E_y_* distribution of the M*_x_*1 mode on the cross-section of the graphene hybrid waveguide, respectively. (**g**,**h**) show the |*E*| and *E_y_* distribution of the M*_x_*′1 mode on the cross-section of the graphene-coated nanowire waveguide, respectively. The white lines in (**e**,**g**) describe the |*E*| distribution of the M*_x_*1 and M*_x_*′1 modes along the *x*-axis direction of the corresponding waveguides, respectively. The “+” and “−” symbols in (**f**,**h**) represent the positive and negative charges, respectively.

The interaction between the two gratings can be adjusted by varying the distance between them. We fixed *R_Si_* as 46 nm and increased the values of *t*_*SiO*_2__ and *p* simultaneously to ensure that *η_o_* was fixed at 0.5. For the S*_x_*′1 mode, *R_i_* = 47 nm is a constant, and *η_i_* decreases with increasing *p*. The resonant wavelength of the S*_x_*′1 mode decreases with decreasing *η_i_*. However, for the S*_x_*1 mode, since *R_o_* increases with increasing *t*_*SiO*_2__, the resonant wavelength increases. Thus, the resonant wavelengths of the two modes exhibit opposite trends with increasing *t*_*SiO*_2__ (Figure 3a).

To better understand the dependence of the resonant wavelength on the interaction between the two gratings, we investigated two other types of graphene gratings without interaction for comparison. The first type of grating is formed by graphene hybrid waveguide [42] array (GHWA) (Figure 3c), and the second type of grating consists of the GCNA (Figure 3d). We refer to the two type of gratings as GHWA and GCNA gratings, which are similar to the outer and inner gratings of Figure 1, respectively. The M*_x_*1 and M*_x_*′1 modes are excited by the GHWA and GCNA gratings (Figure 3e,g), respectively. Both modes are 1-order SCMs, which originate from the quadrupole oscillations in graphene (Figure 3f,h). The occupation rates of the GHWA and GCNA gratings, *η*_1_ and *η*_2_, were set as *η*_1_ = *η_o_* = 0.5 and *η*_2_ = *η_i_* =0.5 − 2(*t*_*SiO*_2__ + *d*)/*p*, respectively. Further, we investigated the impact of the interaction between the inner and outer grating on the resonant wavelengths of the S*_x_*1 and S*_x_*′1 modes. For relatively smaller values of *t*_*SiO*_2__, there are strong interactions between the inner and outer gratings, which cause a more obvious shift of the resonant wavelengths of the S*_x_*1 and S*_x_*′1 modes from the M*_x_*1 and M*_x_*′1 modes, respectively. In addition, the resonant wavelength of the S*_x_*′1 mode is significantly larger than that of the M*_x_*′1 mode because the S*_x_*′1 mode excited by the inner grating is strongly confined within the outer grating. On the contrary, the S*_x_*1 mode excited by the outer grating is subjected to weaker confinement by the inner grating because such a mode can freely transmit outside the waveguide. This results in approximately similar resonant wavelengths of the S*_x_*1 and the M*_x_*1 modes. Therefore, we conclude that for small *t*_*SiO*_2__, the interaction between the two gratings has a greater impact on the resonant wavelength of the S*_x_*′1 mode than that of the S*_x_*1 mode. With increasing *t*_*SiO*_2__, the interaction of the two gratings decreases, the differences between the resonant wavelengths of the S1 and M1 modes and between that of the S*_x_*′1 and M*_x_*′1 modes also gradually decreases, and finally converges on a stable value (Figure 3b).

**Figure 4:**
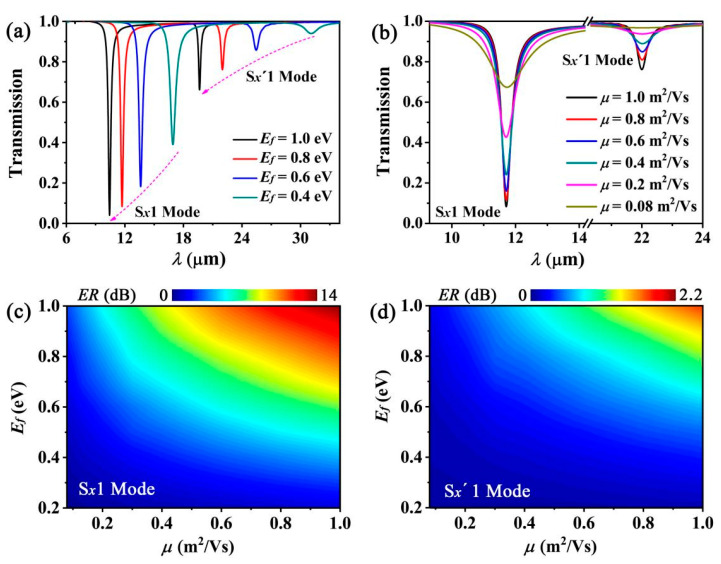
(**a**,**b**) show the simulated transmission spectra at different *E_f_* and *μ* values, respectively. (**c**,**d**) show the extinction ratio (*ER*) of the S*_x_*1 and S*_x_*′1 modes with varying *E_f_* and *μ* values, respectively.

The most intriguing feature of the SPP wave excited by graphene is its tunability. The resonant behaviors of this grating can be adjusted by varying the graphene conductivity, which is primarily determined by the Fermi energy and electron mobility of graphene. The Fermi energy of graphene depends on the carrier concentration, which can be varied by controlling its gate voltage or chemical doping [10]. Efetov et al. [43] reported a significantly high carrier concentration of 4 × 10^14^ cm^−2^ achieved experimentally by using a field-effect transistor (FET) type structure. This carrier concentration value is equivalent to a Fermi energy (*E_f_*) of 1.17 eV. Therefore, we shift *E_f_* from 0.4 eV to 1.0 eV. Previous studies have shown that for a grating formed by the graphene nanoribbon array [24,30] or via GCNA [28], the resonant wavelength is related to *E_f_* via the relation λ∝ (*E_f_*)^−1/2^. As our proposed double graphene gratings are similar to two GCNA gratings, the resonant wavelengths of the S*_x_*1 and S*_x_*′1 modes show a similar trend (blue shift) with increasing *E_f_* (Figure 4a).

Next, we investigated the resonant feature by varying the electron mobility of graphene. An electron mobility of *μ* > 10 m^2^/(Vs) with a peak value (*μ_max_*) of 23 m^2^/(Vs) has been experimentally achieved in suspended exfoliated graphene [44]. Chen et al. reported an experimentally achieved electron mobility as high as 4 m^2^/(Vs) in SiO_2_-supported monolayer graphene [20]. However, since graphene with lower electron mobility is more practical, we selected *μ* values in the range from 0.08 m^2^/(Vs) to 1 m^2^/(Vs). Figure 4b shows that the resonant wavelengths of the two modes are approximately identical for different *μ* values because the resonant behavior is mainly characterized by the imaginary part of conductivity, which is mainly related to the Fermi energy. The optical loss originates from the thermal loss of the current transferring in graphene. Due to the conductivity of graphene *σ_g_* = *ie*^2^*E_f_*/[*πħ*^2^*(ω + iτ*^−1^*)*], *σ_g_* can be expressed as *σ_g_* = *σ_gr_* + *iσ_gi_*, where *σ_gr_* and *σ_gi_* are the real and imaginary parts of the graphene conductivity, respectively. According to Maxwell’s equations, we can obtain

(3) $$\nabla \times \vec{H}=-i(\omega \varepsilon -\sigma_{g\;i})\vec{E}+\sigma_{g\;r}\vec{E}=\vec{J}$$

where $\vec{J}$ is the current density of graphene. The power density of optical loss, *P_J_*, can be derived as

(4) PJ=12Re(J⇀*·E⇀)=12Re[−i(ωε−σgi)E⇀*·E⇀+σgrE⇀*·E⇀]=12σgrE02,

where *E*_0_ is the amplitude of the electric field. Therefore, the optical loss in graphene is determined by the real part of the graphene conductivity, which is related to both the Fermi energy and the electron mobility. On the other hand, we can obtain the optical loss according to the equation *L*[dB/μm] = 8.86 *n_i_k*_0_ [45], where *n_i_* is the imaginary part of the mode effective index. The *n_i_* values can be obtained by the mode solver of the “mode source” from the software Lumerical FDTD Solutions. For example, we can obtain *n_i_* = 0.5863 for the S*_x_*1 mode of the cylindrical LRSPP waveguide with *μ* = 1.0 m^2^/(Vs), *E_f_* = 0.8 eV, *t*_*SiO*_2__ = 12 nm, *R_Si_* = 46 nm, and *λ*_0_ = 10 μm. In this case, the optical loss of this mode is 3.2638 dB/μm. With decreasing *E_f_* and *μ* values, the optical loss in graphene increases, and the notch depth of the transmission spectra decreases. To describe the depth of the notch, we define the extinction ratio (*ER*) of the transmission spectra as *ER* = −10 × log(*T*) (dB), where *T* is the transmission. As can be seen in Figure 4c,d, the *ER* values in both modes increase significantly with increasing *E_f_* and *μ* values. On the other hand, the *ER* value in the S*_x_*1 mode is considerably larger than that in the S*_x_*′1 mode.

**Figure 5:**
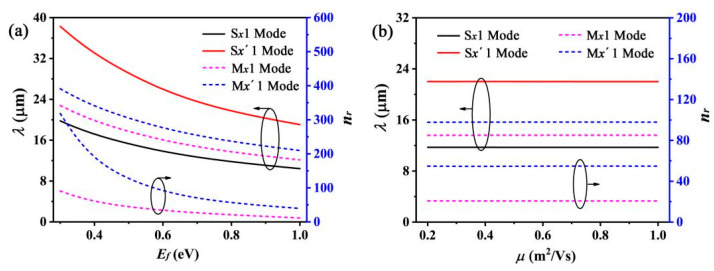
(**a**) Resonant wavelength and effective index as a function of *E_f_* for different modes. (**b**) Resonant wavelength and effective index as a function of *μ* for different modes.

On the other hand, we can also adjust the interaction of the two gratings by varying the graphene characteristics. It can be observed that with decreasing *E_f_,* the resonant wavelengths of the S*_x_*1 and S*_x_*′1 modes exhibit a more obvious shift relative to the M*_x_*1 and M*_x_*′1 modes (Figure 5a). This can be explained as follows. The effective refractive indexes of the M*_x_*1 and M*_x_*′1 modes increase with decreasing *E_f_* (Figure 5a). This improves the confinement of the EM field, and enhances the EM field distributing on the surface of the graphene waveguide. Then, the interaction is strengthened between the two gratings. However, the effective refractive indexes of the M*_x_*1 and M*_x_*′1 modes are almost unchanged with the varying electric mobility of graphene (Figure 5b). This means that the interaction of two gratings will be not changed with the electric mobility. Furthermore, the resonant wavelengths of the S*_x_*1 and S*_x_*′1 modes maintain a stable shift relative to the M*_x_*1 and M*_x_*′1 modes (Figure 5b).

**Figure 6:**
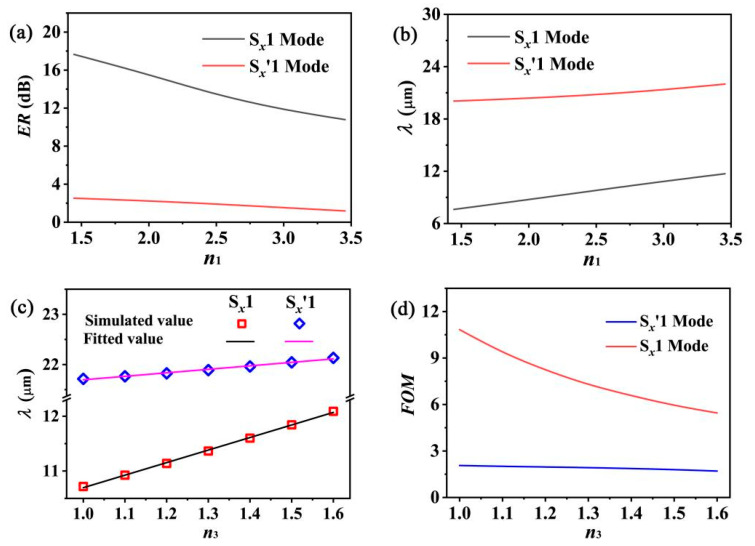
(**a**,**b**) show the *ER* values and the resonant wavelength as a function of *n*_1_ for the two modes, respectively. (**c**,**d**) show the resonant wavelength and the figure of merit (*FOM*) as a function of *n*_3_ for the two modes.

To further improve the *ER* values of the graphene LRSPP waveguide array, a more realistic method is decreasing the refractive index of the dielectric nanowire instead of increasing the *E_f_* and *μ* values. Usually, there is an insignificant loss in dielectric nanowires. However, the EM field confinement is further weakened by decreasing the refractive index of the dielectric nanowire. Subsequently, the interaction between the graphene layer and the EM field is abated. Therefore, the waveguide losses decrease. Figure 6a shows that the *ER* values of the two modes increase approximately 1.7 times when the *n*_1_ values decrease from 3.455 to 1.445. In addition, we showed in our previous study [28] that the resonant wavelength of the GCNA grating is directly proportional to the square root *ε_AVG_* [i.e., *λ* ∝ (*ε_AVG_*)^1/2^], where *ε_AVG_* = (*ε_in_* + *ε_out_*)/2 is the average dielectric constant of the graphene layers, and *ε_in_* and *ε_out_* are the dielectric constant values of the inner and outer layers of graphene, respectively. We observed a similar physical mechanism and phenomena in this study. The resonant wavelengths of the two modes exhibit a red shift with increasing *n*_1_ (Figure 6b). Based on the same principle, the red shift of the resonant wavelengths is also observed with increasing *n*_3_ (Figure 6c). Evidently, the resonant wavelengths of the two modes are in approximately linear relation with *n*_3_. By fitting the simulated data, the two-line equations can be expressed by the following relation for the S*_x_*1 and S*_x_*′1 modes:

*λ*_S*_x_*1_ = 2407.9 × *n*_3_ + 8265 (nm), (5)

*λ*_S*_x′_*1_ = 837.1× *n*_3_ + 20,833.7 (nm) (6)

We can design a sensor to measure the refractive index of the surroundings by using these equations. We usually evaluate the sensing performance of a sensor based on two assessment factors. One is the sensitivity of the wavelength (*S*), which is defined as *S* = Δ*λ/*Δ*n*, where Δ*λ/*Δ*n* is expressed in nanometers per refractive index unit (nm/RIU). The *S* values of the S*_x_*1 and S*_x_*′1 modes are 2407.9 nm/RIU and 837.1 nm/RIU at *n*_1_ = 3.445, respectively. The other assessment factor is the figure of merit (*FOM*) [46,47], which is defined as the ratio of *S* to the full width at half maximum (*FWHM*) of the transmission peak (i.e., *FOM* = *S/FWHM*). The damping of the quadrupole oscillations increases with increasing *n*_3_, which results in an increase in the corresponding *FWHM*. Therefore, the *FOM* values of the two modes decrease with increasing *n*_3_ (Figure 6d). Nevertheless, compared to other graphene-based refractive index sensors [48,49], this sensor exhibits an excellent sensing performance with high *S* and *FOM* values. Most notably, this sensor has an obvious advantage in measuring various gas surroundings with low refractive index values, because the S*_x_*1 mode exhibits higher *FOM* values than the other sensor [50] in this case.

**Figure 7:**
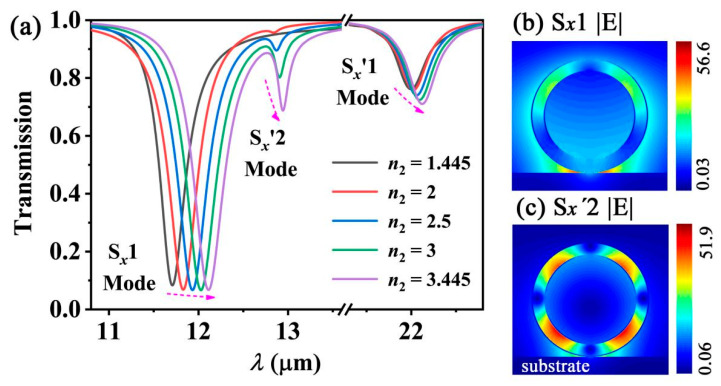
(**a**) Simulated transmission spectra with different *n*_2_. (**b**,**c**) show the |*E*| distribution of the S*_x_*1 and S*_x_*′2 modes, respectively.

The substrate also has an impact on the characteristics of the grating. From Figure 7a, one can find that the resonant wavelengths of the excited modes increase with the refractive index of the substrate *n*_2_. This can be explained as follows. A strong EM field gathers in the interface between the waveguide and the substrate (Figure 7b). This results in the *n_r_* value of the excited mode increasing. According to Equation (2), the resonant wavelength *λ*_0_ can be expressed as *λ*_0_ = *n_r_P/m* for a normal incident light (*θ* = 0°). Thus, the values of *n_r_* and *λ*_0_ increase with *n*_2_. On the other hand, exciting the high order SPP mode becomes easier in the case of increasing *n*_2_. The S*_x_*′2 mode is effectively excited as *n*_2_ = 3.455 (Figure 7a,c).

**Figure 8:**
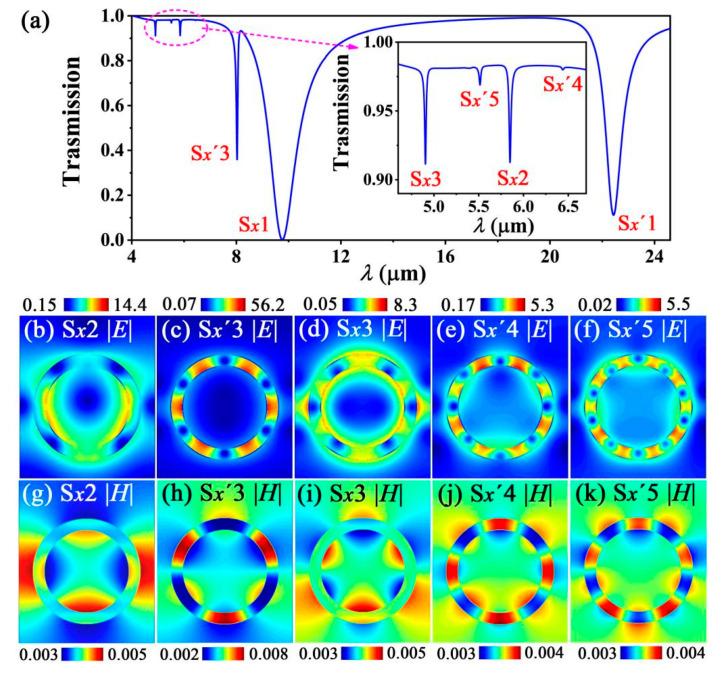
(**a**) Simulated transmission spectra for low and high-order modes. The insert shows the transmission spectra of the region surrounded by the pink short dash line. (**b**–**f**) show the |*E*| distribution of the S*_x_*2, S*_x_*′3, S*_x_*3, S*_x_*′4, and S*_x_*′5 modes, respectively. (**g**–**k**) show the |*H*| distribution of the S*_x_*2, S*_x_*′3, S*_x_*3, S*_x_*′4, and S*_x_*′5 modes, respectively.

Finally, we investigated the excitation approach of the high-order modes. Since the wave vectors of the high-order modes are smaller than that of the low order modes, lower compensation by a grating is required to achieve wave vector matching for higher-order modes. Therefore, a grating with a lower grating constant easily excites higher-order modes. Considering that the *ER* value of the high-order mode is improved with increasing *E_f_* and *μ* and decreasing *n*_1_, we set the parameters of the grating as *R* = 60 nm, *p* = 150 nm, *E_f_* = 1.0 eV, *μ* = 1.0 m^2^/(Vs), and *n*_1_ = 1.72. Consequently, we observed a transmission spectrum with two low-order modes (S*_x_*1 and S*_x_*′1 modes) and five high-order modes (S*_x_*2, S*_x_*′3, S*_x_*3, S*_x_*′4, and S*_x_*′5 modes) (Figure 8a). The |*E*| and |*H*| distributions of the five high-order modes are shown in Figure 8b,c,d,e,f and in Figure 8g,h,i,j,k, respectively. Some high-order modes are missing in this transmission spectra for two reasons. First, some high-order modes, such as the S*_x_*4 and S*_x_*5 modes, cannot be excited using this set of parameters of the grating. Second, although some high-order modes, such as the S*_x_*′2 mode, are excited, their corresponding transmission peaks cannot be observed because their resonant wavelengths are overlapped by the S*_x_*1 mode region.

## 4. Transmission Characteristics of This Grating with an Oblique Incident Light

Finally, we investigate the transmission characteristics of the grating through an oblique incident light of *θ* ≠ 0° (Figure 1b). Usually, two types of modes are used to describe the SPP resonant for an oblique incident light, including the spectral mode (at fixed angle) and the angular mode (at fixed wavelength) [50,51]. According to Equation (2), the resonant wavelength, *λ_res_*, can be expressed as

(7) $$\lambda {\;}_{res}=\frac{\left(n_{r}-\sin\theta \right)p}{m}$$

**Figure 9:**
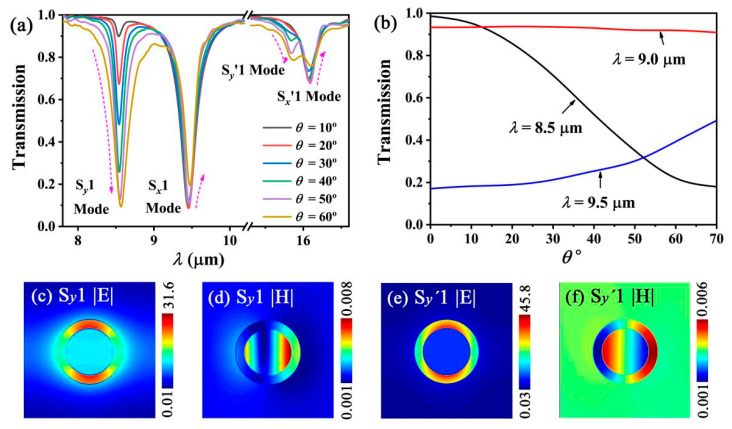
(**a**) Simulated transmission spectra with different incident angular *θ* for an oblique incident light. (**b**) Transmission values as a function of *θ* for the different wavelengths of the incident light. (**c**–**e**) show the |*E*| distribution of the S*_y_*1 and S*_y_*′1 modes, respectively. (**d**–**f**) show the |*H*| distribution of the S*_y_*1 and S*_y_*′1 modes, respectively.

Since the *n_r_* values are much larger than one for the graphene SPP modes, the resonant wavelengths are almost unrelated to *θ*. Figure 9a shows that four SPP modes are excited, and their *λ_res_* values are almost unchanged with varying *θ*. However, the notch depth of the transmission spectra is sensitive to *θ*. This can be explained as follows. The electric field of an oblique incident light can be divided into the two polarization components of *E_x_* and *E_y_*. This results in two types of SPP oscillations along the *x*-axis and the *y*-axis, respectively. Therefore, four 1-order SCMs can be excited through two graphene gratings, as shown in Figure 9a. The S*_x_*1, S*_x_*′1, and S*_y_*1, S*_y_*′1 modes originate from the SPP oscillations along the *x*-axis and the *y*-axis, respectively. The |*E*| and |*H*| distributions of the S*_y_*1, S*_y_*′1 modes are shown in Figure 9c–f, respectively. Upon increasing the incident angular *θ*, the *E_x_* component decreases, while the *E_y_* component increases. The SPP oscillations will be weakened and enhanced along the *x*-axis and the *y*-axis, respectively. Thus, the notch depths of the transmission spectra of the S*_x_*1 and S*_x_*′1 modes decrease, while those of the S*_y_*1 and S*_y_*′1 modes increase, as shown in Figure 9a. Furthermore, the transmission values of the angular mode are almost unchanged with *θ*, when *λ*_0_ is greatly different from *λ_res_*, such as *λ*_0_ = 9.0 μm. However, the transmission values of the angular mode exhibit a significant change with *θ* when *λ*_0_ is close to *λ_res_*, such as *λ*_0_ = 8.5 μm and *λ*_0_ = 9.5 μm (Figure 9b). On the other hand, the resonance angle [50,51] can’t be observed in Figure 9b; this can also be attributed to the fact that the resonant wavelengths are almost unrelated to *θ*.

## 5. Conclusions

In summary, we have proposed and investigated a novel design for a grating, which is composed of a graphene-based cylindrical LRSPP waveguide array. The numerical simulation results demonstrate that two types of SCMs can be excited by a normal incident light passing through this grating, because such a device with double-layer graphene arrays can be equated to two interacting graphene gratings. Therefore, the excited SCMs can be effectively controlled not only by varying the geometrical and physical parameters of the device, but also by changing the interaction between the two graphene gratings. Furthermore, four types of SCMs can be excited by an oblique incident light, which can be divided into two orthogonal beams of light. Based on the transmission features of the SCMs through this grating, several active optical devices, such as filters and refractive index sensors, can be designed. This grating device with double-layer graphene has the potential to excite and manipulate the mid-infrared waves in future photonic integrated circuits.

## Data Availability

The data presented in this study are available on request from the corresponding author.
